# Identification of BRCA1 missense substitutions that confer partial functional activity: potential moderate risk variants?

**DOI:** 10.1186/bcr1826

**Published:** 2007-11-26

**Authors:** Paul K Lovelock, Amanda B Spurdle, Myth TS Mok, Daniel J Farrugia, Sunil R Lakhani, Sue Healey, Stephen Arnold, Daniel Buchanan, kConFab Investigators, Fergus J Couch, Beric R Henderson, David E Goldgar, Sean V Tavtigian, Georgia Chenevix-Trench, Melissa A Brown

**Affiliations:** 1 Queensland Institute of Medical Research, PO Royal Brisbane Hospital, Herston Road, Queensland 4029, Australia; 2 School of Molecular and Microbial Sciences, Coopers Road, University of Queensland, St. Lucia, 4072, Australia; 3 Westmead Millennium Institute, PO Box 412, Westmead, New South Wales, 2145, Australia; 4 Department of Laboratory Medicine and Pathology, Mayo Clinic, 200 First Street SW, Rochester, MN 55905, USA; 5 Molecular & Cellular Pathology, School of Medicine, Herston Road, University of Queensland, 4029, Australia; 6 Peter MacCallum Cancer Centre, St Andrews Place, East Melbourne Victoria, 3002, Australia; 7 Department of Dermatology, University of Utah, 201 Presidents Circle Room, Salt Lake City, UT84112, USA; 8 International Agency for Research on Cancer, 150 Cours Albert-Thomas, 69372 Lyon CEDEX 08, France

## Abstract

**Introduction:**

Many of the DNA sequence variants identified in the breast cancer susceptibility gene *BRCA1 *remain unclassified in terms of their potential pathogenicity. Both multifactorial likelihood analysis and functional approaches have been proposed as a means to elucidate likely clinical significance of such variants, but analysis of the comparative value of these methods for classifying all sequence variants has been limited.

**Methods:**

We have compared the results from multifactorial likelihood analysis with those from several functional analyses for the four BRCA1 sequence variants A1708E, G1738R, R1699Q, and A1708V.

**Results:**

Our results show that multifactorial likelihood analysis, which incorporates sequence conservation, co-inheritance, segregation, and tumour immunohistochemical analysis, may improve classification of variants. For A1708E, previously shown to be functionally compromised, analysis of oestrogen receptor, cytokeratin 5/6, and cytokeratin 14 tumour expression data significantly strengthened the prediction of pathogenicity, giving a posterior probability of pathogenicity of 99%. For G1738R, shown to be functionally defective in this study, immunohistochemistry analysis confirmed previous findings of inconsistent 'BRCA1-like' phenotypes for the two tumours studied, and the posterior probability for this variant was 96%. The posterior probabilities of R1699Q and A1708V were 54% and 69%, respectively, only moderately suggestive of increased risk. Interestingly, results from functional analyses suggest that both of these variants have only partial functional activity. R1699Q was defective in foci formation in response to DNA damage and displayed intermediate transcriptional transactivation activity but showed no evidence for centrosome amplification. In contrast, A1708V displayed an intermediate transcriptional transactivation activity and a normal foci formation response in response to DNA damage but induced centrosome amplification.

**Conclusion:**

These data highlight the need for a range of functional studies to be performed in order to identify variants with partially compromised function. The results also raise the possibility that A1708V and R1699Q may be associated with a low or moderate risk of cancer. While data pooling strategies may provide more information for multifactorial analysis to improve the interpretation of the clinical significance of these variants, it is likely that the development of current multifactorial likelihood approaches and the consideration of alternative statistical approaches will be needed to determine whether these individually rare variants do confer a low or moderate risk of breast cancer.

## Introduction

A significant proportion of inherited breast cancer is caused by mutations in the *BRCA1 *and *BRCA2 *tumour suppressor genes which disrupt their role in cellular DNA repair, cell cycle control, apoptosis, and tumour suppression (reviewed in [1]). Although most mutations that are known to be pathogenic are nonsense or stop mutations and thus are predicted to cause mRNA decay or protein truncation, there are a significant number of missense variants in the *BRCA1 *and *BRCA2 *genes, the clinical consequences of which are unclear [2-5]. It is important that the pathogenicity of these variants be understood, for the benefit of breast cancer patients and their relatives carrying such unclassified variants (UVs) and the clinicians involved in their treatment.

A wide range of approaches for the classification of *BRCA1 *and *BRCA2 *sequence variants have been developed, which include analysis of segregation data, sequence conservation, and protein structure [3-13] and functional analysis based on a range of *in vitro *assays [6-11,14]. Recently, multifactorial likelihood prediction methods have been developed to use data from a variety of sources, including histopathological features of tumours, for the clinical evaluation of UVs [4]. Predictions using this methodology currently rely heavily on data from co-segregation in families and from co-inheritance of variants with known pathogenic mutations in the same gene. Consequently, very rare variants found in single or small families, and also probable pathogenic variants that do not reach the appropriately stringent odds of causality of 1,000:1 suggested for classification as pathogenic, remain formally unclassifiable [4,5]. These findings provide a strong rationale for using functional approaches to contribute additional data to support multifactorial predictions, with the caveat that such approaches are most useful for assessment of variants located in known functional domains for which *in vitro *functional assays have been developed.

Building on our previous studies, we selected four UVs for additional analyses, including tumour immunohistochemistry using markers known to be associated with *BRCA1 *mutation status [12], and *in vitro *assays to examine the effects on BRCA1 function. The study included one variant we had previously classified as pathogenic by multifactorial likelihood analysis (G1738R) and three variants that remained unclassified after multifactorial analysis (R1699Q, A1708V, and A1708E) [3-5]. The A1708E variant acted as a positive control for functional assays since we and others [3,9,13] have previously shown this variant to be functionally compromised. All four variants map to the transcriptional activation domain (TAD) and the putative interaction site for RNA polymerase II, RNA helicase A, and multiple transcription factors [1]. We present our comparison of multifactorial likelihood predictions of pathogenicity and functional analysis of these BRCA1 variants.

## Materials and methods

### Tumour characterisation and revised multifactorial analysis

#### Patient recruitment and consent

As described previously [5], pedigrees with UVs in *BRCA1 *and *BRCA2 *were ascertained by the Kathleen Cuningham Foundation Consortium for Research into Familial Breast Cancer (kConFab) according to eligibility criteria established by the organisation [15,16]. With informed consent from participants, breast tumour sections from archival pathology specimens were recalled for research studies. This research study was approved by the human research ethics committees of the Peter MacCallum Cancer Centre, the Queensland Institute of Medical Research, and the University of Queensland.

#### Pathology review

Criteria for classifying tumours as '*BRCA1*-like' or 'not *BRCA1*-like' have been described previously [5].

#### Tumour immunohistochemistry

Oestrogen receptor (ER), cytokeratin 5/6 (CK5/6), and cytokeratin 14 (CK14) immunohistochemistry was carried out as described previously [12].

#### Tumour microsatellite instability

Ten microsatellite markers (BAT25, BAT26, BAT40, BAT34C4, D5S346, D17S250, ACTC, D18S55, D10S197, and MYCL) were analysed for microsatellite instability (MSI) status according to a previously established protocol [17]. Tumour tissue was compared with normal tissue. Tumourclassification was as follows: MSI-high if three or more markers demonstrated instability, MSI-low if one or two markers demonstrated MSI, and MSI-stable if no marker exhibited MSI.

#### Prior probability of pathogenicity from amino acid conservation, and location of the mutation in specific known functional domains

Missense substitutions and in-frame deletions were classified according to their location within one of two recognised functional domains of the proteins, the C terminus region containing the BRCA1 BRCT repeats, defined loosely as amino acids 1,396 to 1,862, and the BRCA2 DNA-binding domain (amino acids 2,500 to 3,098). Variants were also categorised according to whether the wild-type residue involved in the substitution/deletion was evolutionarily conserved through to the pufferfish *Tetraodon*, using multiple sequence alignments available on the Web site [18]. Heterogeneity analysis of 1,433 variants in the Myriad Genetic Laboratories, Inc. (Salt Lake City, UT, USA) database was used to estimate the proportion of Myriad deleterious variants in three classifications [19]: (a) invariant position in BRCA1 C terminus domain/BRCA2 DNA-binding domain (BRCT/DBD) domain, proportion = 0.73; (b) variable position in BRCT/DBD domain, proportion = 0.08; and (c) position outside of BRCT/DBD domain, proportion = 0.02. The values were then used as prior probabilities of being deleterious for the classification of the studied variants. All variants in this study fell within the BRCA1 BRCT domain.

#### Co-occurrence with pathogenic mutations

We queried the Myriad Genetic Laboratories, Inc. database of approximately 100,000 full-sequence tests to determine the number of times a UV was observed, and the number of *different *deleterious mutations observed to co-occur with each variant, as a measure of the number of times the UV is seen *in trans *with a deleterious mutation. Phase of the variant and mutation was established for a subset of individuals. Observations for variants were excluded if *in cis *with a mutation, included if *in trans *with a mutation, and assumed to be *in trans *with at least *n *- 1 observations for *n *observations with different deleterious mutations of unknown phase.

#### Histopathology

Available invasive tumour sections were analysed for parameters recognised to be associated with *BRCA1 *mutation status [12,20,21]. Immunohistochemistry scoring was performed as described previously [12]. Scoring was performed by a single pathologist (SRL).

#### Pedigree causality analysis

Bayes factor analysis of families was performed as described previously and incorporated no information additional to that published previously [3,5].

#### Derivation of probabilities and multifactorial likelihood scoring

Probabilities were derived for each of the components included in the study, under the assumption that each factor was independent. For the co-occurrence component, we estimated the likelihood that any given UV was causal, as described previously [4]. The Bayes factor was included directly as a likelihood ratio (LR) score for the pedigree analysis component. Tumour expression of ER, CK5/6, and CK14 was used for calculating histopathological LR scores, based on the previously reported prevalence of the combined immunotypes of these independent predictors of *BRCA1 *mutation status in breast tumours [12]. The likelihoods for causality were ER-positive (irrespective of cytokeratin score) = 0.14:1; negative for all three markers = 0.87:1; ER-negative, CK14-negative, CK5/6-positive = 5.6:1; ER-negative, CK14-positive, CK5/6-negative = 2.6:1; and ER-negative, CK14-positive, CK5/6-positive = 27.4:1. For the single ER-negative grade 3 tumour with insufficient material available for cytokeratin analysis, the likelihood was calculated based on ER expression and grade (LR 2.95:1), as described previously [5].

The individual LRs were multiplied to calculate an overall multifactorial LR, assuming statistical independence of the sources of information. Bayes rule was then used to calculate a posterior probability that the variant was deleterious from the multifactorial LR and the prior probability as determined by sequence alignment.

### Functional analysis

#### *BRCA1 *cDNA plasmids

For the transcriptional activation assays, the pGal4B vector and pGal4B vector containing a cDNA sequence encoding C-terminal residues 1,528 to 1,863 (571 amino acids) of *BRCA1 *containing TADs 1 and 2 [22] were kindly donated by Jane Visvader (Walter and Eliza Hall Institute of Medical Research, Melbourne, Australia). For generating control templates for single nucleotide primer extension (SNuPE) assays and for the cytoplasmic localisation and centrosome amplification assays, a pZeoSV plasmid containing the full-length BRCA1 cDNA with or without UV [3] was used.

#### Generation of mutagenised *BRCA1 *cDNA plasmids

Mutations in *BRCA1 *cDNA plasmids were introduced using a polymerase chain reaction (PCR)-mediated mutagenesis protocol as described previously [3] using *Pfu *Turbo Taq polymerase (Invitrogen Corporation, Carlsbad, CA, USA) and the following primers incorporating the appropriate sequence change (underlined, bold): BRCA1 1699Q forward 5'-gat gct gag ttt gtg tgt gaa c**a**g aca ctg aaa tat ttt cta gg-3', BRCA1 1699Q reverse 5'-cct aga aaa tat ttc agt gtc **t**gt tca cac aca aac tca gca tc-3', BRCA1 1708V forward 5'-ctg aaa tat ttt cta gga att g**t**g gga gga aaa tgg gta gtt ag-3', BRCA1 1708V reverse 5'-cta act acc cat ttt cct ccc **a**ca att cct aga aaa tat ttc ag-3', BRCA1 1738R forward 5'-gag cat gat ttt gaa gtc aga **a**ga gat gtg gtc aat gga aga aac-3', and BRCA1 1738R reverse 5'-gtt tct tcc att gac cac atc tc**t** tct gac ttc aaa atc atg ctc-3'. Mutagenic primers used to generate the BRCA1 1708E variant are described elsewhere [3]. Mutagenised clones were confirmed by sequencing, and large-scale DNA preps were made using commercial preparation kits (Qiagen Inc., Valencia, CA, USA).

#### Cell culture

Lymphoblastoid cell lines (LCLs) were grown in RPMI with 10% foetal calf serum (FCS) and antibiotic/antimycotic (Gibco-BRL, now part of Invitrogen Corporation). 293T cells were cultured in Dulbecco's modified Eagle's medium (DMEM) with 10% FCS and antibiotic/antimycotic. T47D cells were cultured in RPMI media with 10% FCS, 10 μg/mL insulin, and antibiotic/antimycotic. All cells were incubated at 37°C in 5% CO_2_.

#### RNA extraction from cell lines

RNA was extracted from cell lines using Trizol Reagent (Invitrogen Corporation) according to the manufacturer's instructions and DNAse treated using the Ambion DNA-Free kit (Ambion, Inc., Austin, TX, USA). cDNA was made from RNA using the Invitrogen Superscript III Reverse Transcriptase kit according to the manufacturer's instructions and used directly in PCR.

#### Single nucleotide primer extension assays

Templates for the SNuPE assay were generated by reverse transcription-PCR of RNA extracted from LCLs and PCR of the matching control plasmids (wild-type and mutagenised BRCA1) using either of the primers described previously [3] for the R1699Q, A1708V, and G1738R variants. PCR products from wild-type and mutagenised BRCA1 control templates and LCL cDNAs were then used in the SNuPE assay as described previously [3] using the following primers: SNuPE1699For 5'-gat gct gag ttt gtg tgt gaa c-3', SNuPE1699Rev 5'-cct aga aaa tat ttc agt gtc-3', SNuPE1708F 5'-ctg aaa tat ttt cta gga att g-3', SNuPE1708R 5'-gct aac tac cca ttt tcc tcc c-3', SNuPE 1738F 5'-gag cat gat ttt gaa gtc aga-3', and SNuPE 1738R 5'-ttc ttc cat tga cca cat ctc-3'. Radiolabelled products were resolved on a denaturing polyacrylamide gel and visualised by autoradiography, as described previously [3].

#### Tryptic digestion profiles

Plasmids carrying the mutagenised BRCA1 cDNA or wild-type BRCA1 cDNA (1,571 base-pair product encompassing exons 12 to 24) were used as templates to generate PCR products with primers described previously [3] for the R1699Q, A1708V, and G1738R variants. The PCR products were transcribed and translated *in vitro *using the Promega TNT Coupled Reticulocyte Lysate System and sulfur-35 L-methionine (PerkinElmer, Melbourne, Australia) according to the manufacturer's instructions. Protein products were digested in increasing concentrations of trypsin and resolved on a 14% acrylamide gel. Products were visualised using autoradiography or the Typhoon™ Phosphorimaging system (Amersham Biosciences, now part of GE Healthcare, Little Chalfont, Buckinghamshire, UK).

#### Foci formation assays

MCF-7 human breast cancer cells were maintained in DMEM supplemented with 10% FCS and grown at 37°C in a humidified 5% CO_2 _atmosphere. Cells were seeded onto sterile glass coverslips and transfected at 50% to 60% confluency with 2 to 5 μg of plasmid DNA using Lipofectamine Reagent (Invitrogen Corporation) according to the manufacturer's instructions. At 6 hours after transfection, the transfection mix was removed and replaced with DMEM containing 10% FCS. At 44 hours after transfection, cells were either left untreated or exposed to 15 Gy of radiation from a cesium-137 source (Gammacell 1000 irradiator; Atomic Energy of Canada Limited, Mississauga, ON, Canada) and then allowed to recover at 37°C for 4 hours. Cells were fixed in 3.7% formalin/PBS for 15 minutes, permeabilised in 0.2% Triton-PBS for 10 minutes, and processed for immunostaining. Myc-tagged ectopic BRCA1 was detected by immunofluorescence using the anti-Myc rabbit polyclonal antibody A-14 (Santa Cruz Biotechnology, Inc., Santa Cruz, CA, USA). Myc antibody was detected with anti-rabbit Alexa Fluor 594 (Invitrogen Corporation). YFP-BARD1 was co-transfected to ensure nuclear localisation of BRCA1 isoforms. Cell nuclei were counterstained with the chromosome dye Hoechst 33285 (Sigma-Aldrich, St. Louis, MO, USA). The intranuclear foci localisation of each ectopic protein was determined by scoring cells using an Olympus BX40 epifluorescence microscope (Olympus, Tokyo, Japan), and the proportion of cells displaying 0, 1 to 10, or greater than 10 nuclear foci per cell was determined as previously described [7]. Digital images were collected using a SPOT camera. *P *values were determined using two-tailed *t *tests.

#### Centrosome amplification assays

293T cells were cultured on glass coverslips and transfected with Myc-BRCA1 wild-type and mutant constructs using Fugene 6 (Roche, Melbourne, Australia) according to the manufacturer's instructions. For indirect immunofluorescence, cells were fixed with cold methanol, permeabilised, and stained with primary anti-centrin-2 (1:800) polyclonal (MC1, kindly provided by Jeffrey Salisbury) and anti-Myc (9E10) (1:200) monoclonal (Santa Cruz Biotechnology, Inc.) antibodies 96 hours after transfection. Alexa 568 goat anti-mouse and 488 goat anti-rabbit secondary antibodies were subsequently added, along with 1 μg/mL Hoechst (Molecular Probes Inc., now part of Invitrogen Corporation). Centriole numbers were counted in a minimum of 100 Myc-expressing cells from each of two independent experiments using a Zeiss LSM510 confocal microscope (Carl Zeiss, Jena, Germany).

#### BRCA1 transcriptional activation domain reporter assays

Human 293T and T47D cells were transiently transfected with 0.5 μg of the pG5CAT reporter plasmid and 1 μg of the pGal4B plasmids described above in triplicate in six-well plates using Fugene 6 according to the manufacturer's instructions. Cell lysates were assayed for chloramphenicol acetyltransferase (CAT) activity using the Roche CAT enzyme-linked immunosorbent assay kit. CAT activity was normalised to total cell extract protein assayed by the Bio-Rad Protein Assay reagent (Bio-Rad Laboratories, Inc., Hercules, CA, USA). *P *values were determined using two-tailed *t *tests.

## Results

A description of the variants under study is shown in Table 1. Based on our previously reported odds for causality using the multifactorial likelihood analysis approach [5], together with the established thresholds of greater than 1,000:1 for pathogenicity and less than 1:100 for neutrality [4], G1738R was classified at the start of this study as pathogenic, whereas R1699Q, A1708V, and A1708E were considered unclassified. To further investigate the potential clinical significance of these variants using the likelihood approach, we analysed the tumours arising in carriers of these variants for ER and cytokeratin expression in order to extend the histopathology component of the model and we updated and revised data for the co-inheritance and sequence conservation components of the model. We also performed a range of previously described assays for BRCA1 function.

**Table 1:** Revised multifactorial likelihood analysis of *BRCA*1 unclassified variants

BRCA1 variant	Previous multifactorial likelihood classification: odds for causality^a^	Evolutionary conservation to *Tetraodon *and in BRCT domain	Sequence alignment prior probability^b^	Myriad frequency (*in trans *with deleterious mutation)	LR from co-occurrence with a deleterious mutation	Segregation analysis: Bayes factor	Tumour histology (number analysed)	Grade	ER	CK 5/6	CK14	LR pathology	Multifactorial likelihood odds: co-occurrence, segregation, and pathology	Posterior probability of a variant being deleterious
BRCA1 5215 G>A (R1699Q)	141:1	Invariant, to *Tetraodon*, inside BRCT	0.73	33 (0)	3.010	1.043	Not BRCA1-like (1)	1	Pos	Neg	Neg	0.14	0.440	0.543
BRCA1 5242 C>T (A1708V)	41:1	Invariant, to *Tetraodon*, inside BRCT	0.73	6 (0)	1.220	0.227	BRCA1-like (1)	3	Neg	NA	NA	2.95	0.817	0.668
BRCA1 5242 C>A (A1708E)	262:1	Invariant, to *Tetraodon*, inside BRCT	0.73	76 (0)	12.700	4.470	BRCA1-like (1)	3	Neg	Pos	Pos (focal)	27.38	1554.024	0.999
BRCA1 5331 G>A (G1738R)	5871:1	Invariant, to *Tetraodon*, inside BRCT	0.73	8 (0)	1.300	20.250	BRCA1-like (1)	3	Neg	Neg	Pos (strong)	0.37	9.724	0.963
							Not BRCA1-like (1)	0	Pos	Neg	Neg			

^a^Chenevix-Trench and colleagues [5] and Lovelock and colleagues [3]; ^b^Easton and colleagues [19]. CK, cytokeratin; ER, estrogen receptor; LR, likelihood ratio; NA, not applicable; Neg, negative; Pos, positive.

### Tumour characterisation and revised multifactorial analysis

We revised and extended our previous likelihood analysis as described in Materials and methods. Specifically, sequence conservation scores were interpreted as a prior probability that incorporated amino acid position in known functional domains of BRCA1 and BRCA2 [19], likelihoods for co-inheritance were from an updated dataset, segregation analysis was as performed previously [3,5], and an amended histopathological likelihood was derived from either ER and grade as in Chenevix-Trench and colleagues [5] or ER and CK5/6 and CK14 as described in Materials and methods. Incorporating this additional information gave posterior probabilities of pathogenicity of 54.3% for R1699Q, 68.8% for A1708V, 99.9% for A1708E, and 96.3% for G1738R (Table 1).

As detailed in Table 1, the data indicate that evolutionary analysis supported pathogenicity for all variants. Since no variants co-occurred with deleterious mutations in the Myriad dataset, the co-occurrence likelihoods provided relatively little information, with the exception of A1708E, which is observed many times in this dataset. Segregation analysis was most useful for G1738R. Assessment of histological and immunohistochemical features of tumours from UV carriers showed that the single tumour from an R1699Q carrier was not associated with any tumour features commonly observed in *BRCA1 *high-risk mutation carriers. The single tumours from carriers of A1708V and A1708E were *BRCA1*-like, with the expected immunohistochemical features. Tumours from two carriers of the G1738R variant differed from each other in most respects, with only one displaying features characteristic of *BRCA1 *tumours. Interestingly, even the cytokeratin expression profile for the tumour with *BRCA1*-like morphological features was only moderately suggestive of a causative mutation in the *BRCA1 *gene (LR 2.6:1).

As an additional assessment of tumour characteristics, we conducted MSI analysis of tumours since the BRCA1 BRCT domain is known to bind to MLH1 and it has been suggested previously that mutations in the BRCA1 BRCT domain may cause mismatch repair deficiency [8]. Furthermore, limited evidence from MSI analysis of a single tumour carrying a BRCA1 W1837R variant has been interpreted to suggest that missense mutations in the BRCT domain may present with a microsatellite-unstable tumour phenotype [8]. However, our analysis of tumour DNA from the two G1738R carriers showed that both tumours were microsatellite-stable.

### Functional analysis of *BRCA1 *unclassified variants

**Figure 1:**
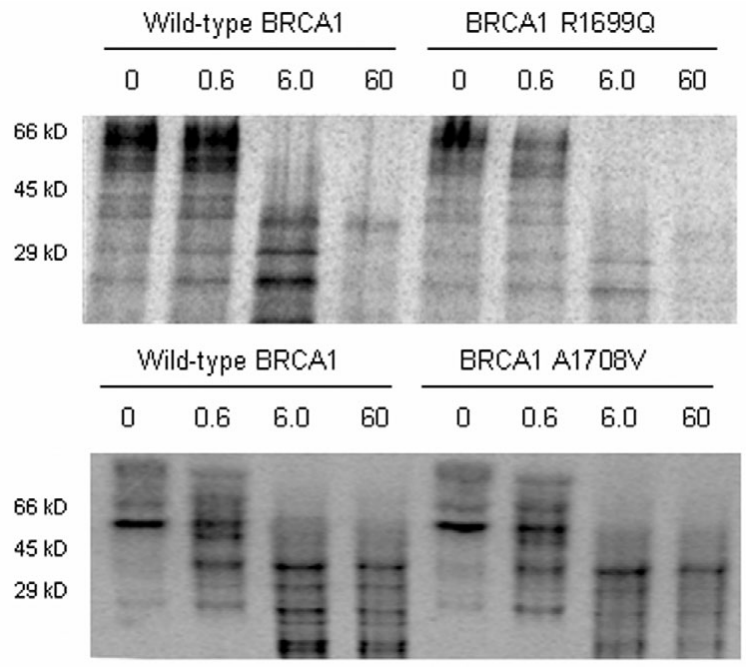
BRCA1 R1699Q causes destabilisation of the BRCT domain. *In vitro*-transcribed and -translated BRCA1 cDNA fragments containing wild-type or unclassified variant sequence, incorporating sulfur-35-labelled methionine, were treated with increasing concentrations of trypsin (μg/mL) and resolved on SDS-PAGE.

**Figure 2:**
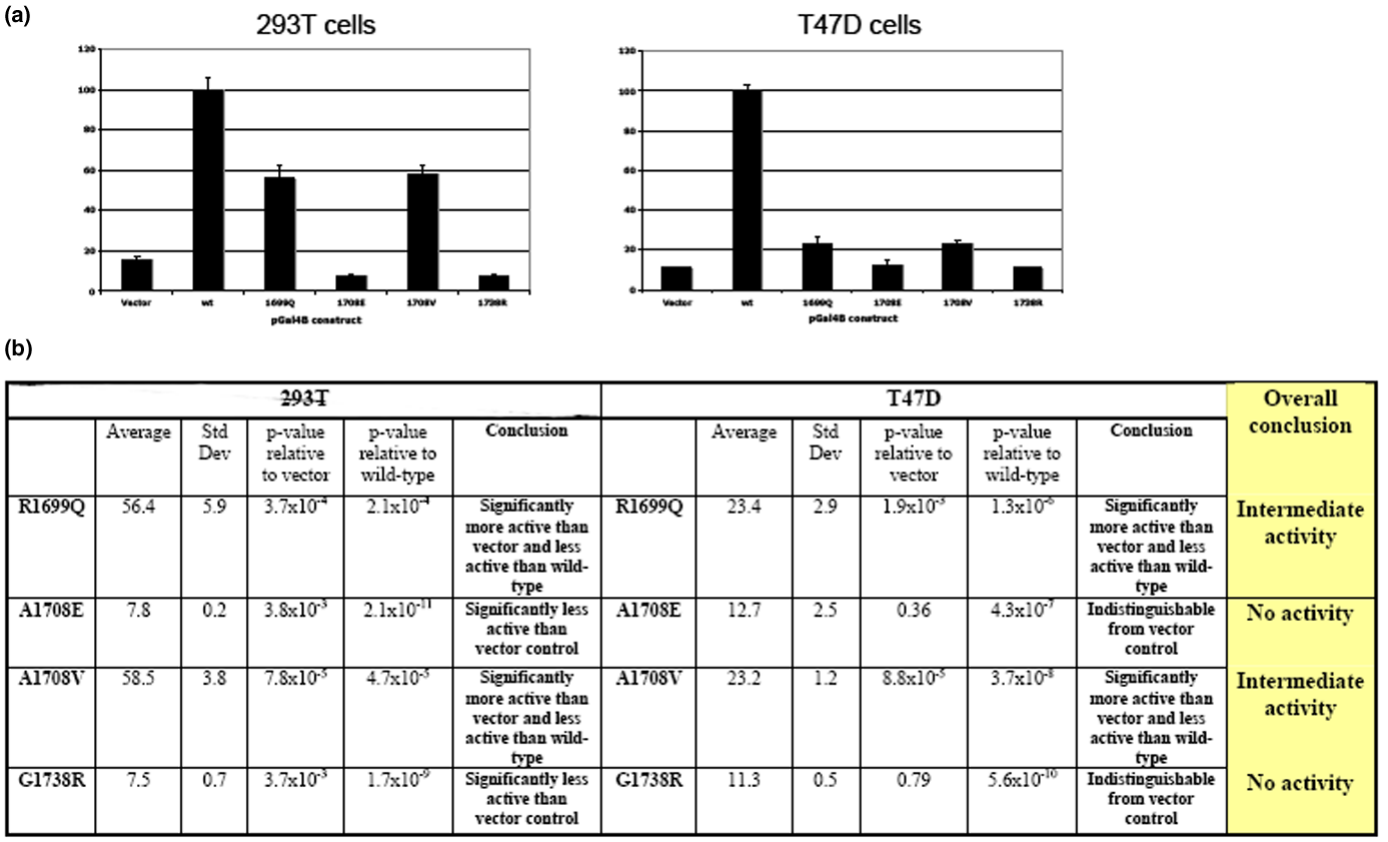
Transcriptional transactivation by C-terminal BRCA1 variants. **(a) **Graphical representation of results. Values are the mean and standard deviation of triplicate transfections. **(b) **Table showing means, standard deviations, and *p *values relative to vector and wild-type (wt) controls.

**Figure 3:**
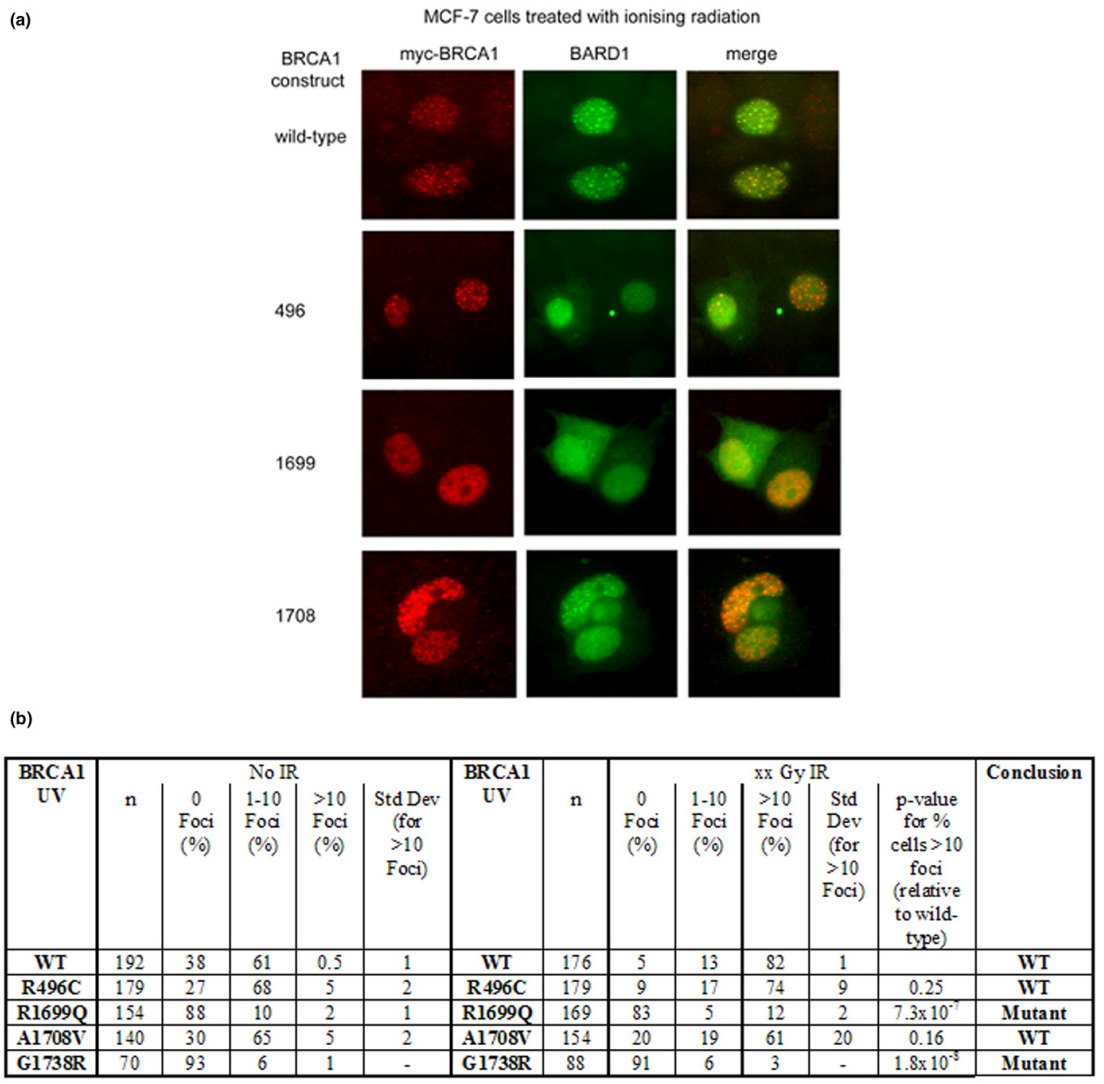
Varied capacity of C-terminal BRCA1 variants to form foci in response to DNA damage. **(a) **Post-ionising radiation (IR) nuclear foci formation in cells co-transfected with the nuclear chaperone BARD1. **(b) **Summary of nuclear foci formation data in untreated cells and cells treated with IR. UV, unclassified variant; WT, wild-type.

**Figure 4:**
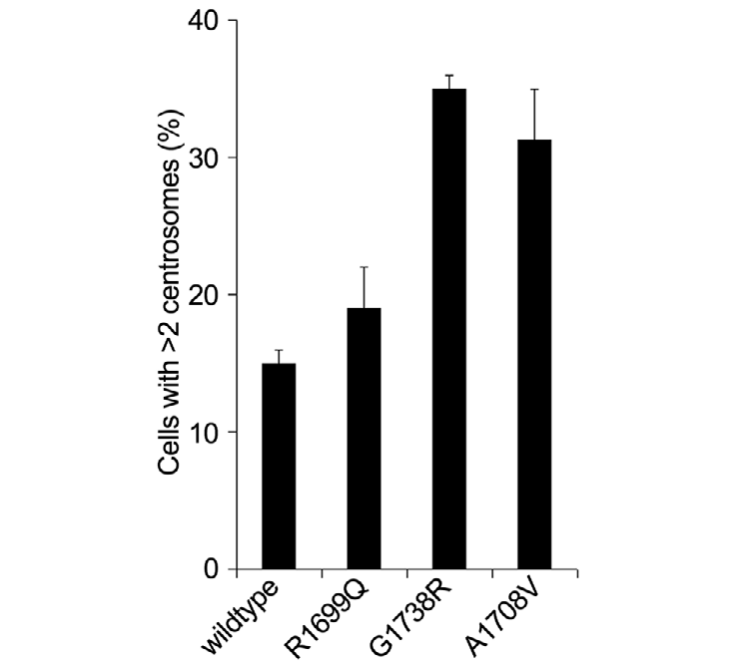
Centrosome amplification. Graphical representation of percentage of cells transfected with BRCA1 wild-type and variants that display centrosome amplification (greater than two centrosomes). Values are the mean and standard error of duplicate studies.

To further characterise these variants, a range of functional assays were performed (Figures 1 to 4 and summarised in Table 2). As single nucleotide changes can affect mRNA and protein stability, we first determined the levels of the products of gene expression from the UV allele, using SNuPE on LCLs from carriers of each variant and *in vitro *transcription and translation of cDNA constructs carrying these variants, respectively. SNuPE analysis revealed no bias in expression levels of either the wild-type or UV alleles in LCLs isolated from *BRCA1 *UV carriers (data not shown). Similarly, *in vitro *transcription and translation assays revealed no change in the levels of protein product (Figure 1, zero dose). Thus, these variants do not appear to effect either mRNA or protein stability.

**Table 2:** Summary of functional analyses on *BRCA1 *unclassified variants

BRCA1 variant	Expression (RNA and protein)	BRCT structure (trypsin sensitivity)	Transcription activation activity	Nuclear foci formation	Centrosome amplification	Conclusion
BRCA1 5215 G>A (R1699Q)	Wild-type	Destabilised	Intermediate activity	Defective	Wild-type	Intermediate on 1 and defective on 2 of 5 criteria
BRCA1 5242 C>T (A1708V)	Wild-type	Wild-type	Intermediate activity	Normal	Positive	Intermediate on 1 and defective on 1 of 5 criteria
BRCA1 5242 C>A (A1708E)	Wild-type	Destabilised	No activity	Defective^a^	Positive	Defective on 4 of 5 criteria
BRCA1 5331 G>A (G1738R)	Wild-type	Destabilised	No activity	Defective	Positive	Defective on 4 of 5 criteria

^a^Results from assays performed in Lovelock and colleagues [3].

Amino acid substitutions in the BRCT domain of BRCA1 have previously been shown to destabilise its structure and this is associated with an increased susceptibility to trypsin-mediated proteolysis [23,24]. Whereas previous studies have shown this to be the case for G1738R [24] and A1708E [3], the effect of R1699Q and A1708V on BRCT structure has not been investigated. To address this, we carried out trypsin digestion assays on radioactively labelled *in vitro*-translated products derived from fragments of the *BRCA1 *cDNA. In the proteolytic assays, the *in vitro*-translated products encoding the A1708V variant showed tryptic digestion profiles similar to those of the wild-type product at all trypsin concentrations tested, whereas the protein products of R1699Q, G1738R, and A1708E were degraded at and above 6 μg/mL of trypsin (Figure 1 and data not shown). These results confirm previously published data on A1708E and G1738R and indicate that the BRCT domain of R1699Q, but not A1708V, is destabilised.

Several assays were carried out using an expression construct encoding the BRCA1 TAD to ascertain any effects of R1699Q, A1708V, and G1738R on the capacity of the region to activate a co-transfected reporter construct in two independent mammalian cell lines (Figure 2). BRCA1 A1708E was also included as a UV known to be inactive in transcriptional transactivation activity [3]. The G1738R and A1708E variants abolished reporter activity to levels comparable to or below those of the empty control vector. However, the A1708V and R1699Q variants appeared to have an intermediate effect on reporter activity. The magnitude of this effect was different between the 293T cell line and the T47D cell line, with the levels of reporter activity being approximately 55% of the wild-type in the 293T cell line and approximately 20% of the wild-type in the T47D cell line.

Cancer-associated mutations in *BRCA1 *can prevent nuclear localisation of BRCA1 and its contribution to nuclear DNA repair foci formation in response to DNA damage (for example, by ionising radiation [IR]) [6,7]. To determine whether the UVs under study caused mislocalisation of BRCA1 and/or defects in nuclear foci formation and therefore were likely to be pathogenic, we transfected MCF-7 cells with the expression constructs and measured foci formation and cytoplasmic localisation. As expected, wild-type BRCA1 and a known benign variant (R496C) showed a strong increase in IR-inducible nuclear foci (Figure 3). A similar, albeit variable, response was seen in cells expressing BRCA1 A1708V. Although the average percentage of cells containing greater than 10 foci is lower than in cells expressing wild-type BRCA1, this difference was not statistically significant (Figure 3b). In contrast, cells expressing R1699Q and G1738R showed a significantly diminished response, as we have reported previously for A1708E using the same assay [3]. Together, these results suggest that R1699Q, G1738R, and A1708E cause a defect in the localisation and/or DNA damage response of BRCA1 whereas A1708V does not.

BRCA1 is involved in the regulation of centrosomes, and some cancer-associated mutations are associated with centrosome amplification [3,25]. We assayed centrosome amplification in cells transfected with expression constructs carrying the UVs to determine whether these UVs induced centrosome amplification similarly to known deleterious mutations [3]. Centrosome amplification in cells expressing BRCA1 R1699Q was not significantly different from amplification in cells expressing wild-type BRCA1 (Figure 4 and Table 2). In contrast, centrosomes were more frequently amplified in cells expressing BRCA1 A1708V, G1738R, and A1708E, the latter in agreement with our previously published observations [3].

The results of the functional analyses of the variants investigated in this study are summarised in Table 2. There was no evidence to suggest that the variants may directly affect mRNA or protein expression. A1708E and G1738R proteins were defective in all other assays performed. In contrast, R1699Q was defective for two assays and had intermediate function for one assay (out of four assays), and A1708V had intermediate function for the TAD assay, defective function in a centrosome amplification assay, and normal function in a nuclear foci formation assay. This suggests that R1699Q and A1708V may confer partial BRCA1 activity.

### Summary of results

A comparison of the conclusions from the original multifactorial likelihood analysis, the revised multifactorial analysis incorporating revised evolutionary and co-occurrence components and additional tumour features, and the functional data are shown in Table 3. This comparison confirms previous reports that BRCA1 A1708E is pathogenic. It provides functional evidence of pathogenicity for G1738R which is supported by the revised multifactorial analysis. The latter includes updated and improved sequence conservation analysis and tumour immunohistochemical analysis but has lost some power over previous analyses due to the necessary exclusion of loss of heterozygosity data. This comparison also shows that the R1699Q and A1708V variants, with only a moderate probability of association with a high risk of cancer, are both at least partially functionally compromised.

**Table 3:** Summary of results

Variant	Original odds for causality^a^	Original conclusion	Posterior probability of pathogenicity from revised multifactorial likelihood analysis (Table 1)	Functional data (Table 2)	Conclusion
BRCA1 R1699Q	141:1	UV	54.3%	Intermediate on 1 and defective on 2 of 5 criteria	Possible low to moderate risk
BRCA1 A1708V	41:1	UV	68.8%	Intermediate on 1 and defective on 1 of 5 criteria	Possible low to moderate risk
BRCA1 A1708E	262:1	UV	99.9%	Defective on 4 of 5 criteria	High risk
BRCA1 G1738R	5,871:1	Pathogenic	96.3%	Defective on 4 of 5 criteria	High risk

^a^Chenevix-Trench and colleagues [5]. UV, unclassified variant.

## Discussion

Missense amino acid substitutions in BRCA1 and BRCA2 are difficult to interpret. Multifactorial likelihood models incorporating a range of information therefore have been developed to enable predictions to be made regarding their causality. The underlying assumptions of these models are based on information derived from classical high-risk mutation carriers and predominantly truncating mutations, which currently classify variants as either high-risk or of low clinical significance/neutral. These models are therefore of clinical utility for predicting the pathogenicity of the small subset of variants that are associated with a high risk of disease. The ability of this approach to distinguish between variants that are truly neutral and those associated with a low risk of cancer is limited. Moreover, the final predictions of pathogenicity are necessarily driven by the availability of information on segregation and tumour phenotype (numbers of families and tumours available) and thus it is difficult to assess whether apparent UVs may actually be associated with a moderate risk of cancer. In addition, the model assumes that missense mutations exhibit the same characteristics (cancer risk and tumour phenotypes) as observed for truncating mutations.

The results of this study show for the first time that incorporating data on the immunohistochemical characteristics of tumours arising in carriers of these variants can sometimes improve the prediction of pathogenicity. In the case of BRCA1 A1708E, which was previously classified as a UV using multifactorial likelihood analysis based on limited data, revised multifactorial analysis yielded a posterior probability of pathogenicity of 99%. Tumour data contributed considerably to this classification, with the presence of basal cytokeratin markers and the absence of ER staining consistent with the tumour phenotype of a defective *BRCA1 *gene. Together with the functional data for this variant, the results provide evidence that this missense variant exhibits the features of a classical pathogenic *BRCA1 *mutation.

Our results also emphasise the fact that multifactorial predictions must be viewed in light of the available data and the underlying assumptions of the model, particularly the assumption that *BRCA1 *missense mutation carriers are expected to display classical '*BRCA1*-like' features. Our previous analysis of tumour immunohistochemical features of UV carriers suggested disparate results for two tumours from G1738R carriers, a result we confirm here with more detailed immunohistochemical analysis of basal markers of the BRCA1 mutation phenotype. While it is possible that some tumours occurring in BRCA1 carriers may be sporadic tumours not driven by abnormalities in BRCA1, the ages at onset of the two G1738R carriers (44 and 43 years) do not obviously point to the possibility of a sporadic tumour on a BRCA1 mutation background as an explanation for the observed data. It remains to be tested whether missense mutation carriers will display features similar to truncating mutations, but this will presumably require a large collaborative effort given the paucity of known pathogenic missense mutations. However, we are cautiously optimistic that at least a subset of BRCA1 missense carriers will be identifiable from histopathological characteristics. We have observed that two of the three tumours from carriers of functionally abrogated BRCA1 alleles display BRCA1-like features. Moreover, access to unpublished core data from the kConFab sample set has identified that breast tumours from carriers of RING-finger domain mutations with available pathology data (nine tumours) are all high-grade and those with known receptor status (four tumours) are all ER-negative/progesterone receptor-negative. It is important to note that our previous pathogenic classification for G1738R was not driven by immunohistopathology results but rather by the observation of loss of the wild-type allele in both BRCA1 G1738R tumours studied, data we excluded in this study due to the observation that our loss of heterozygosity data on classified variants do not support the underlying assumptions of the model. Nevertheless, the revised multifactorial data support the observation that this variant exhibits the same functional deficiencies as the A1708E variant, with a 96% posterior probability of pathogenicity. This also supports other studies that report G1738R as a founder Greek mutation [26], with reported odds of 11,470:1 for causality from analysis of seven Greek families [27].

Importantly, our study has highlighted the fact that the multifactorial approach will require development to assess whether a variant is associated with a low or moderate risk of cancer. Revised multifactorial analysis incorporating tumour features did not strongly support a high risk for either R1699Q or A1708V, with posterior probabilities perhaps suggestive of a moderately increased risk. Although this may appear to agree with the fact that functional assays suggest that *both *variants are at least partially functionally comprised, it should be noted that the posterior probabilities were driven by the sequence alignment component of the analysis. It is likely that the individual components of the multifactorial approach may have different predictive power to assess variants that are associated with a lower risk of cancer, and development of the current multifactorial model and/or alternative statistical approaches will need to be considered to test the hypothesis that variants of intermediate function may be associated with an intermediate risk of cancer.

In this study, we did not include analysis of tumour loss of heterozygosity as a component of the multifactorial analysis, as done previously [5], since interrogation of published and unpublished data we have generated for a range of UVs has revealed increased loss of the variant compared with that expected for the underlying hypothesis used to calculate likelihood estimates, irrespective of their final classification. This suggests that the underlying assumptions for this component may not be appropriate for the classification of missense variants.

We also carried out a range of assays for the variants under study to provide novel and supporting data toward a more comprehensive description of the functional defects, if any, associated with these variants. Results from functional analyses of variants are largely supported by sequence alignment and protein modelling predictions. Sequence alignment and BRCA1 R1699Q maps to the N-terminal BRCT motif of the TAD suggest that it is deleterious, whereas modelling and *in vitro *proteolytic assays indicate that the variant may affect the structure or conformation of the BRCT domain [23]. Functional analysis of this variant suggests that it may partially compromise BRCA1 function. TAD assays in 293T and T47D mammalian cells indicated an intermediate phenotype, 56% of wild-type in 293T cells and 23% of wild-type in T47D cells (Figure 4). This intermediate activity is consistent with similar analyses performed by Vallon-Christersson and colleagues [9], who showed approximately 20% activity of R1699Q in 293T cells. The difference in the level of activity may reflect differences in the cDNA construct and reporter system used. In other assays, R1699Q was either defective (nuclear foci formation) or indistinguishable from wild-type (centrosome amplification). Taken together, these findings suggest that this variant causes a significant yet incomplete loss of BRCA1 function.

The A1708V variant is also located in the N-terminal BRCT motif of BRCA1. Replacement of an alanine residue with a valine at this position is predicted to affect the conformation of BRCA1 by causing an incompatibility with a bend structure in the helix [5] and it is possible that this predicted change may have functional effects. However, to date, no functional data have been presented for this variant. Here, we showed that A1708V possesses reduced transcriptional transactivation activity in two independent cell lines, similar to the R1699Q variant, and that it induces centrosome amplification. In contrast, this variant caused no significant change in nuclear foci formation.

The G1738R is located in the interval between the N- and C-terminal BRCT motifs. This induces a conformational change in BRCA1 which renders it susceptible to tryptic digestion [23], and a recent report of transcriptional activity from assays in yeast and mammalian cells indicates pathogenicity for this variant [28]. This is supported by results from our mammalian transcription transactivation assays, in which we observed a reduced transactivation capacity of G1738R similar to that of the deleterious variant A1708E. In addition, we have shown that nuclear foci formation and centrosome amplification are compromised to levels similar to that observed for the A1708E missense variant, commonly considered to be pathogenic [3]. Collectively, current data from functional analysis of the G1738R variant reported here and elsewhere [28] suggest that it exhibits functional characteristics of a true pathogenic mutation.

Our functional analysis of the A1708E TAD variant confirms our previous findings [3] that this variant is deleterious. In addition, we have extended our previous multifactorial likelihood analysis and now provide convincing evidence for pathogenicity using this approach, with a posterior probability of pathogenicity of 99%.

The overall results from multifactorial and functional analyses highlight the known limitation of multifactorial analysis in that it was not designed to distinguish between variants that are truly neutral and those associated with a moderate or low risk of cancer. The need to address or circumvent this limitation is obvious from the increasing number of reports of low to moderate risk genetic variants contributing to breast cancer [29-33] and recent evidence that rare variants of known breast cancer genes (including *BRCA1 *and *BRCA2*) act additively or multiplicatively to significantly increase cancer risk at the individual level [34].

Our multifactorial analysis indicates that both R1699Q and A1708V are not *high-risk *variants, but functional analyses have shown that each displays either intermediate or defective phenotype in some but not all assays (Table 2), raising the possibility that these variants may be associated with a low to moderate risk of cancer. Interestingly, whereas both variants display intermediate activity with respect to transcriptional transactivation, only R1699Q appears defective in nuclear foci formation and only A1708V induced centrosome amplification. Foci formation is known to be dependent on an intact BRCT structure [7], and our results using trypsin sensitivity analysis of BRCT structure are consistent with this, with R1699Q displaying BRCT destabilisation and defective foci formation whereas A1708V is normal on both counts. Our results also suggest that transcriptional transactivation activity is likely to involve a region of the C terminus other than that targeted by trypsin digestion and suggest that the domains involved on foci formation and the regulation of centrosomes are likely to be independent. Given that these specific functional assays to some extent examine discrete activities of the BRCA1 protein, it would be preferable in the shorter term to use a battery of tests to assess altered function. However, given that the ultimate aim of an effective functional test is to have the minimum number of robust test results, ultimately an assay that measures general tumour suppressor activity is required. Such an assay would be expected to reflect the contribution of multiple overlapping and independent activities and also to establish whether loss of a single activity may be sufficient to disrupt its overall function as a tumour suppressor. Unfortunately, although the most reliable functional assays may improve estimates of the level or type of compromised function, they are unlikely at this stage to provide a direct translation to measures of cancer risk. An alternative study design such as large collaborative case-control studies [35], or pooled family studies assessing risk associated with variant of similar functional capacity, may be required to provide better estimates of cancer risk associated with variants of intermediate functional phenotype.

## Conclusion

Our results suggest that the addition of tumour immunohistochemical expression can add value to the classification of likely causal *BRCA1 *variants using multifactorial likelihood analysis and that functional assays are a useful adjunct to multifactorial analysis. We also show that use of data from a range of assays may be required to identify variants of moderately reduced function, and we suggest that modified or alternative statistical approaches will be required to assess whether such variants are associated with a low to moderate risk of cancer.

## Abbreviations

BRCT/DBD = BRCA1 C terminus domain/BRCA2 DNA-binding domain; CAT = chloramphenicol acetyltransferase; CK5/6 = cytokeratin 5/6; CK14 = cytokeratin 14; DMEM = Dulbecco's modified Eagle's medium; ER = oestrogen receptor; FCS = foetal calf serum; IR = ionising radiation; kConFab = Kathleen Cuningham Foundation Consortium for Research into Familial Breast Cancer; LCL = lymphoblastoid cell line; LR = likelihood ratio; MSI = microsatellite instability; PCR = polymerase chain reaction; SNuPE = single nucleotide primer extension; TAD = transcriptional activation domain; UV = unclassified variant.

## Competing interests

The authors declare that they have no competing interests.

## Authors' contributions

PKL carried out experimental work and drafted the manuscript. ABS secured funding for the study, participated in project conception and supervision of experiments, carried out the multifactorial analysis, and contributed substantially to the drafting of the manuscript. MTSM, FJC, DJF, and BRH carried out or supervised aspects of the experimental work. SRL, SH, SA, and DB participated in the tumour characterisation component of the study. DEG and SVT provided data and advice regarding multifactorial analysis. GC-T participated in project conception and project supervision and critically reviewed the manuscript. MAB secured funding for the project, participated in project conception, supervised the functional arm of the study, and contributed substantially to the drafting of the manuscript. All authors approved the final manuscript. PKL and ABS contributed equally to this work.

## References

[B1] Boulton SJ (2006). Cellular functions of the BRCA tumour-suppressor proteins. Biochem Soc Trans.

[B2] Deffenbaugh AM, Frank TS, Hoffman M, Cannon-Albright L, Neuhausen SL (2002). Characterization of common BRCA1 and BRCA2 variants. Genet Test.

[B3] Lovelock PK, Healey S, Au W, Sum EY, Tesoriero A, Wong EM, Hinson S, Brinkworth R, Bekessy A, Diez O (2006). Genetic, functional, and histopathological evaluation of two C-terminal BRCA1 missense variants. J Med Genet.

[B4] Goldgar DE, Easton DF, Deffenbaugh AM, Monteiro AN, Tavtigian SV, Couch FJ (2004). Integrated evaluation of DNA sequence variants of unknown clinical significance: application to BRCA1 and BRCA2. Am J Hum Genet.

[B5] Chenevix-Trench G, Healey S, Lakhani S, Waring P, Cummings M, Brinkworth R, Deffenbaugh AM, Burbidge LA, Pruss D, Judkins T (2006). Genetic and histopathologic evaluation of BRCA1 and BRCA2 DNA sequence variants of unknown clinical significance. Cancer Res.

[B6] Rodriguez JA, Au WW, Henderson BR (2004). Cytoplasmic mislocalization of BRCA1 caused by cancer-associated mutations in the BRCT domain. Exp Cell Res.

[B7] Au WW, Henderson BR (2005). The BRCA1 RING and BRCT domains cooperate in targeting BRCA1 to ionizing radiation-induced nuclear foci. J Biol Chem.

[B8] Quaresima B, Faniello MC, Baudi F, Crugliano T, Cuda G, Costanzo F, Venuta S (2006). *In vitro *analysis of genomic instability triggered by BRCA1 missense mutations. Hum Mutat.

[B9] Vallon-Christersson J, Cayanan C, Haraldsson K, Loman N, Bergthorsson JT, Brøndum-Nielsen K, Gerdes AM, Møller P, Kristoffersson U, Olsson H (2001). Functional analysis of BRCA1 C-terminal missense mutations identified in breast and ovarian cancer families. Hum Mol Genet.

[B10] Coyne RS, McDonald HB, Edgemon K, Brody LC (2004). Functional characterization of BRCA1 sequence variants using a yeast small colony phenotype assay. Cancer Biol Ther.

[B11] Ostrow KL, McGuire V, Whittemore AS, DiCioccio RA (2004). The effects of BRCA1 missense variants V1804D and M1628T on transcriptional activity. Cancer Genet Cytogenet.

[B12] Lakhani SR, Reis-Filho JS, Fulford L, Penault-Llorca F, van der Vijver M, Parry S, Bishop T, Benitez J, Rivas C, Bignon YJ (2005). Prediction of BRCA1 status in patients with breast cancer using estrogen receptor and basal phenotype. Clin Cancer Res.

[B13] Phelan CM, Dapic V, Tice B, Favis R, Kwan E, Barany F, Manoukian S, Radice P, van der Luijt RB, van Nesselrooij BP (2005). Classification of BRCA1 missense variants of unknown clinical significance. J Med Genet.

[B14] Scully R, Ganesan S, Vlasakova K, Chen J, Socolovsky M, Livingston DM (1999). Genetic analysis of BRCA1 function in a defined tumor cell line. Mol Cell.

[B15] Eligibility criteria for recruitment of families into KConFaB – 'Daylesford Criteria'. http://www.kconfab.org/Collection/Eligibility.shtml.

[B16] Mann GJ, Thorne H, Balleine RL, Butow PN, Clarke CL, Edkins E, Evans GM, Fereday S, Haan E, Gattas M (2006). Analysis of cancer risk and BRCA1 and BRCA2 mutation prevalence in the kConFab familial breast cancer resource. Breast Cancer Res.

[B17] Lindor NM, Burgart LJ, Leontovich O, Goldberg RM, Cunningham JM, Sargent DJ, Walsh-Vockley C, Petersen GM, Walsh MD, Leggett BA (2002). Immunohistochemistry versus microsatellite instability testing in phenotyping colorectal tumors. J Clin Oncol.

[B18] Align GVGD. http://agvgd.iarc.fr/.

[B19] Easton DF, Deffenbaugh AM, Pruss D, Frye C, Wenstrup RJ, Allen-Brady K, Tavtigian SV, Monteiro AN, Iversen ES, Couch FJ (2007). A systematic genetic assessment of 1,433 sequence variants of unknown clinical significance in the BRCA1 and BRCA2 breast cancer-predisposition genes. Am J Hum Genet.

[B20] Lakhani SR, Gusterson BA, Jacquemier J, Sloane JP, Anderson TJ, van de Vijver MJ, Venter D, Freeman A, Antoniou A, McGuffog L (2000). The pathology of familial breast cancer: histological features of cancers in families not attributable to mutations in BRCA1 or BRCA2. Clin Cancer Res.

[B21] Lakhani SR, Jacquemier J, Sloane JP, Gusterson BA, Anderson TJ, van de Vijver MJ, Farid LM, Venter D, Antoniou A, Storfer-Isser A (1998). Multifactorial analysis of differences between sporadic breast cancers and cancers involving BRCA1 and BRCA2 mutations. J Natl Cancer Inst.

[B22] Sum EY, Peng B, Yu X, Chen J, Byrne J, Lindeman GJ, Visvader JE (2002). The LIM domain protein LMO4 interacts with the cofactor CtIP and the tumor suppressor BRCA1 and inhibits BRCA1 activity. J Biol Chem.

[B23] Williams RS, Chasman DI, Hau DD, Hui B, Lau AY, Glover JN (2003). Detection of protein folding defects caused by BRCA1-BRCT truncation and missense mutations. J Biol Chem.

[B24] Williams RS, Glover JN (2003). Structural consequences of a cancer-causing BRCA1-BRCT missense mutation. J Biol Chem.

[B25] Lingle WL, Barrett SL, Negron VC, D'Assoro AB, Boeneman K, Liu W, Whitehead CM, Reynolds C, Salisbury JL (2002). Centrosome amplification drives chromosomal instability in breast tumor development. Proc Natl Acad Sci USA.

[B26] Konstantopoulou I, Rampias T, Ladopoulou A, Koutsodontis G, Armaou S, Anagnostopoulos T, Nikolopoulos G, Kamakari S, Nounesis G, Stylianakis A Greek BRCA1 and BRCA2 mutation spectrum: two BRCA1 mutations account for half the carriers found among high-risk breast/ovarian cancer patients. Breast Cancer Res Treat.

[B27] Anagnostopoulos T, Pertesi M, Konstantopoulou I, Armaou S, Kamakari S, Nasioulas G, Athanasiou A, Dobrovic A, Young MA, Goldgar D G1738R is a BRCA1 founder mutation in Greek breast/ovarian cancer patients: evaluation of its pathogenicity and inferences on its genealogical history. Breast Cancer Res Treat.

[B28] Carvalho MA, Marsillac SM, Karchin R, Manoukian S, Grist S, Swaby RF, Urmenyi TP, Rondinelli E, Silva R, Gayol L (2007). Determination of cancer risk associated with germ line BRCA1 missense variants by functional analysis. Cancer Res.

[B29] Rahman N, Seal S, Thompson D, Kelly P, Renwick A, Elliott A, Reid S, Spanova K, Barfoot R, Chagtai T (2007). PALB2, which encodes a BRCA2-interacting protein, is a breast cancer susceptibility gene. Nat Genet.

[B30] Seal S, Thompson D, Renwick A, Elliott A, Kelly P, Barfoot R, Chagtai T, Jayatilake H, Ahmed M, Spanova K (2006). Truncating mutations in the Fanconi anemia J gene BRIP1 are low-penetrance breast cancer susceptibility alleles. Nat Genet.

[B31] Steffen J, Varon R, Mosor M, Maneva G, Maurer M, Stumm M, Nowakowska D, Rubach M, Kosakowska E, Ruka W (2004). Increased cancer risk of heterozygotes with NBS1 germline mutations in Poland. Int J Cancer.

[B32] Steffen J, Nowakowska D, Niwinska A, Czapczak D, Kluska A, Piatkowska M, Wisniewska A, Paszko Z (2006). Germline mutations 657del5 of the NBS1 gene contribute significantly to the incidence of breast cancer in Central Poland. Int J Cancer.

[B33] Karppinen SM, Erkko H, Reini K, Pospiech H, Heikkinen K, Rapakko K, Syvaoja JE, Winqvist R (2006). Identification of a common polymorphism in the TopBP1 gene associated with hereditary susceptibility to breast and ovarian cancer. Eur J Cancer.

[B34] Johnson N, Fletcher O, Palles C, Rudd M, Webb E, Sellick G, Dos Santos Silva I, McCormack V, Gibson L, Fraser A (2007). Counting potentially functional variants in BRCA1, BRCA2 and ATM predicts breast cancer susceptibility. Hum Mol Genet.

[B35] Cox A, Dunning AM, Garcia-Closas M, Balasubramanian S, Reed MW, Pooley KA, Scollen S, Baynes C, Ponder BA, Chanock S (2007). A common coding variant in CASP8 is associated with breast cancer risk. Nat Genet.

